# Enrolment characteristics associated with retention among HIV negative Kenyan gay, bisexual and other men who have sex with men enrolled in the *Anza Map* *ema* cohort study

**DOI:** 10.1002/jia2.25598

**Published:** 2020-10-01

**Authors:** Colin Kunzweiler, Robert C Bailey, Duncan O Okall, Susan M Graham, Supriya D Mehta, Boaz Otieno‐Nyunya, Gaston Djomand, Fredrick O Otieno

**Affiliations:** ^1^ Division of Epidemiology and Biostatistics School of Public Health University of Illinois at Chicago Chicago Illinois USA; ^2^ UNIM Research and Training Centre Nyanza Reproductive Health Society Kisumu Kenya; ^3^ Departments of Medicine Global Health, and Epidemiology University of Washington Seattle Washington USA; ^4^ Division of Global HIV/AIDS Centers for Disease Control and Prevention Kisumu Kenya; ^5^ Division of Global HIV/AIDS and Tuberculosis Centers for Disease Control and Prevention Atlanta Georgia USA

**Keywords:** gay and bisexual men who have sex with men, GBMSM, care and treatment, cohort study, missed follow‐up visits, retention, HIV, HIV negative, Kenya

## Abstract

**Introduction:**

Most gay, bisexual and other men who have sex with men (GBMSM) live in rights‐constrained environments making retaining them in research to be as hard as recruiting them. To evaluate *Anza Mapema*, an HIV risk‐reduction programme in Kisumu, Kenya, we examined the enrolment sociodemographic, behavioural, psychosocial and clinical factors associated with missing two or more follow‐up visits for GBMSM participating in Anza Mapema.

**Methods:**

Between August 2015 and November 2017, GBMSM were enrolled and followed in a prospective cohort study with quarterly visits over 12 months. At enrolment, men were tested for HIV and sexually transmitted infections and completed questionnaires via audio computer‐assisted self‐interview. Because the Kenya Ministry of Health recommends HIV testing every three to six months for GBMSM, the retention outcome in this cross sectional analysis was defined as missing two consecutive follow‐up visits (vs. not missing two or more consecutive visits). Multivariable logistic regression estimated the adjusted odds ratios (aOR) and 95% confidence intervals (CI) for the associations of the enrolment characteristics with the binary outcome of retention.

**Results and discussion:**

Among 609 enrolled HIV‐negative GBMSM, the median age was 23 years (interquartile range, 21 to 28 years), 19.0% had completed ≤8 years of education and 4.1% had resided in the study area <1 year at enrolment. After enrolment, 19.7% missed two consecutive follow‐up visits. In the final multivariable model, the odds of missing two consecutive follow‐up visits were higher for men who: resided in the study area <1 year at enrolment (aOR, 4.14; 95% CI: 1.77 to 9.68), were not living with a male sexual partner (aOR, 1.59; 95% CI: 1.01 to 2.50), and engaged in transactional sex during the last three months (aOR, 1.70; 95% CI: 1.08 to 2.67).

**Conclusions:**

One in five men missed two consecutive follow‐up visits during this HIV prevention study despite intensive retention efforts and compensation for travel and participation. Participants with recent community arrival may require special support to optimize their retention in HIV prevention activities. Live‐in partners of participants may be enlisted to support greater engagement in prevention programmes, and men who engage in transactional sex will need enhanced counselling and support to stay in longitudinal studies.

## 1 INTRODUCTION

Studies conducted throughout sub‐Saharan Africa demonstrate that gay, bisexual and other men who have sex with men (GBMSM) have HIV prevalence rates two to four times higher than the general male population [1] with substantial stigma due to criminalization of their sexual practices [2, 3]. To address their increased risk ‐of HIV acquisition and transmission, it is necessary to design, implement and test scalable comprehensive HIV prevention and treatment interventions tailored to the needs of GBMSM [4, 5, 6, 7], and to identify the challenges and opportunities involved in engaging and retaining participants in studies and prevention programmes [8]. We conducted a longitudinal cohort study called *Anza Mapema* (Kiswahili for “Start Early”) whose purpose was to optimize regular HIV testing, linkage to care and retention in HIV prevention and care among GBMSM in Kisumu, Kenya. In this analysis, we sought to identify enrolment factors associated with non‐retention of HIV‐negative GBMSM in the *Anza Mapema* study, which may translate to improved retention practices.

## 2 METHODS

### 2.1 Study population

Recruitment into the *Anza Mapema* Cohort study occurred from August 2015 to September 2016 using snowball methodology [9]. Eligibility criteria were as follows: age ≥18 years, self‐reported anal or oral intercourse with another man in the previous six months, not participating in another HIV intervention or vaccine study, and residing in the study area (within Kisumu County) [10]. Of the 711 men completing enrolment procedures in *Anza Mapema*; 75 were HIV‐positive at baseline, 14 seroconverted, 4 died and 9 withdrew from the study during follow‐up, leaving 609 in this analysis. Men who withdrew from the study or died were excluded from this analysis because they may not have had sufficient time to experience the primary outcome; HIV‐positive GBMSM and men who seroconverted were excluded from analysis because their follow‐up was monthly. The *Anza Mapema* study was approved by the ethical review boards of Maseno University, the University of Illinois at Chicago and the University of Washington.

### 2.2 Study procedures

Following provision of written informed consent, all men provided detailed locator information, completed an audio computer‐assisted self‐interview (ACASI), underwent HIV counselling and testing, completed a medical history and physical examination and provided specimens for sexually transmitted infections testing at enrolment. The same procedures were followed at quarterly follow‐up visits for 12 months. Participants were referred for additional services including alcohol and substance abuse and psychological counselling by study personnel as necessary or as requested.

### 2.3 Retention strategies

As part of retention, personnel established and regularly communicated with a community advisory board throughout recruitment and follow‐up. Personnel and peer outreach workers created and maintained a social media account (https://www.facebook.com/NRHSAnzaMapema/) promoting the delivery of HIV prevention and treatment services to GBMSM in the study. They also hosted social activities, including movie nights, support groups and religious services, four to five days per week. Personnel conducted case reviews of specific participants with suboptimal visit attendance and developed strategies for improving retention.

To minimize missed follow‐up visits, participants received a reminder card with the date of their next visit at the end of each visit. The study offered flexible hours including early morning, evening and weekend appointments. A second clinic site was opened in an office building in the centre of Kisumu City to enhance confidentiality for men who did not want to attend the main study site. Participants were compensated 500 Kenyan shillings (US$5) for their time and travel costs at each quarterly study visit. For participants who relocated from the study area within the country and wanted to continue participation, transportation was arranged and paid for by the study.

Personnel collected names, nicknames, physical address, primary telephone number, e‐mail address and social media identity for each study participant, and a map was drawn of the participant’s neighbourhood and directions to his home. The name and location of frequent hangouts were recorded, and contact information of family members and close friends were recorded with the participant’s approval.

Personnel obtained permission to contact participants via multiple modalities including telephone calls, SMS text messaging, WhatsApp, Facebook messaging and in‐person contacts. Visit reminders were sent at the beginning of each participant’s visit window period, mid‐way in the visit window period and the day before the scheduled visit. Up to three reminders were sent at each time point via multiple modalities. The participant was called up to three times the day following a missed visit and again during the first and second week following the missed visit. If necessary, study personnel initiated up to three attempts of physical tracing at the participant’s address.

### 2.4 Study retention definition

A study visit was classified as “missed” if the participant did not attend his visit within 1.5 months before or 1.5 months after his scheduled visit date. We considered men who had missed any two consecutive follow‐up visits as not retained in the *Anza Mapema* study. This outcome aligns with the National AIDS and STI Control Programme of the Kenya Ministry of Health, which recommends HIV testing among key populations every three to six months [11].

### 2.5 Predictor variables

All sociodemographic, behavioural and psychosocial predictor variables included in this analysis were collected via ACASI (Questionnaire Development Software version 3.0, NOVA Research Company, Silver Spring, MD, USA) at enrolment. ACASI questionnaires were available in DhoLuo, Kiswahili and English. All scales and the cut‐offs applied for behavioural and psychosocial variables are presented elsewhere [10].

### 2.6 Statistical analysis

The analysis is cross sectional with the outcome being two consecutive missed visits. Differences between baseline explanatory variables and outcome were assessed by chi‐square test for categorical variables. Variables with *p* < 0.20 by likelihood ratio testing were entered in multivariable logistic regression, with backwards stepwise selection, retaining variables with *p* < 0.05 by likelihood ratio test.

## 3 RESULTS AND DISCUSSION

### 3.1 Study population

The baseline sociodemographic, behavioural and psychosocial characteristics of the participants are shown in Table 1.

**Table 1:** Distribution of sociodemographic, behavioural and psychosocial characteristics collected at baseline among HIV‐negative GBMSM in Kenya by retention status (N = 609)

Variable	Missed 2 consecutive visits		Total
	No	Yes	
	N = 489 (80.3%)	N = 120 (19.7%)	N = 609 (100%)
Sample	n (%)^a^	n (%)^a^	n (%)^b^
Age, years (median [IQR])	23 (21 to 28)	23 (21 to 26)	23 (21 to 28)
Age, years			
18 to 24	279 (78.8)	75 (21.2)	354 (58.1)
≥25	210 (82.3)	45 (17.7)	255 (41.9)
Education, years			
0 to 8	91 (78.5)	25 (21.5)	116 (19.1)
9 to 12	249 (79.3)	65 (20.7)	314 (51.6)
≥13	149 (83.2)	30 (16.8)	179 (29.4)
Currently enrolled in school			
No	334 (79.3)	87 (20.7)	421 (69.1)
Yes	155 (82.5)	33 (17.5)	188 (30.9)
Employment status			
Less than full‐time employed	224 (78.9)	60 (21.1)	284 (46.6)
Full‐time employed	265 (81.5)	60 (18.5)	325 (53.4)
Uncertain/Very uncertain financial status			
No	132 (78.6)	36 (21.4)	168 (27.6)
Yes	357 (81.0)	84 (19.0)	441 (72.4)
Tribe/ethnicity			
Other	84 (76.4)	26 (23.6)	110 (18.1)
Luo	405 (81.2)	94 (18.8)	499 (81.9)
Resided in Kisumu for less than 1 year			
No	458 (81.1)	107 (18.9)	565 (92.8)
Yes	13 (54.2)	11 (45.8)	24 (3.9)
Missing	18 (90.0)	2 (10.0)	20 (3.3)
Any religious affiliation			
No	35 (81.4)	8 (18.6)	43 (7.1)
Yes	454 (80.2)	112 (19.8)	566 (92.9)
Marital status			
Single	361 (79.9)	91 (20.1)	452 (74.2)
Married or living with female partner	53 (81.5)	12 (18.5)	65 (10.7)
Separated or divorced from female partner	75 (81.5)	17 (18.5)	92 (15.1)
Gay or homosexual sexual identity			
No	149 (78.8)	40 (21.2)	189 (31.0)
Yes	340 (80.9)	80 (19.1)	420 (69.0)
Currently living with a male sexual partner			
No	306 (78.1)	86 (21.9)	392 (64.4)
Yes	183 (84.3)	34 (15.7)	217 (35.6)
Transactional sex (participant gave or received money, food, or housing) during the last three months			
No	188 (83.2)	38 (16.8)	226 (37.1)
Yes	301 (78.6)	82 (21.4)	383 (62.9)
Ever had sex with a female partner			
No	138 (82.1)	30 (17.9)	168 (27.6)
Yes	351 (79.6)	90 (20.4)	441 (72.4)
Always used condoms during AI with a male sexual partner (last three months)			
No	279 (79.5)	72 (20.5)	351 (57.6)
Yes	198 (81.5)	45 (18.5)	243 (39.9)
Did not have anal sex	12 (80.0)	3 (20.0)	15 (2.5)
Usual sexual position during sex with a male partner			
Receptive or versatile	209 (81.0)	49 (19.0)	258 (42.4)
Insertive	271 (79.5)	70 (20.5)	341 (56.0)
Missing	9 (90.0)	1 (10.0)	10 (1.6)
Experienced recent trauma due to same‐sex behaviours (last two weeks) (USAID HPI)			
No	192 (80.7)	46 (19.3)	238 (39.1)
Yes	250 (79.6)	64 (20.4)	314 (51.5)
Missing	47 (82.5)	10 (17.5)	57 (9.4)
Experienced sexual or physical abuse during childhood (CECA)			
No	104 (86.7)	16 (13.3)	120 (19.7)
Yes	381 (78.7)	103 (21.3)	484 (79.5)
Missing	4 (80.0)	1 (20.0)	5 (0.8)
Harmful alcohol use (AUDIT ≥ 8)			
No	242 (79.6)	62 (20.4)	304 (49.9)
Yes	247 (81.3)	57 (18.7)	304 (49.9)
Missing	0 (0.0)	1 (100.0)	1 (0.2)
Harmful substance use (DAST ≥ 3)			
No	375 (80.6)	90 (19.4)	465 (76.4)
Yes	114 (79.2)	30 (20.8)	144 (23.6)
Any injection drug use in last year			
No	458 (80.1)	114 (19.9)	572 (93.9)
Yes	31 (83.8)	6 (16.2)	37 (6.1)
Social support (MOS‐SS; continuous range: 0 to 100 scale; median [IQR])	50 (34 to 66)	50 (36 to 61)	50 (36 to 64)
Moderately severe or severe depressive symptoms (PHQ‐9 ≥ 15)			
No	440 (80.7)	105 (19.3)	545 (89.5)
Yes	49 (76.6)	15 (23.4)	64 (10.5)
Circumcision status			
No	121 (83.5)	24 (16.5)	145 (23.8)
Yes	368 (79.3)	96 (20.7)	464 (76.2)
STI status			
Negative for CT and/or NG	420 (80.5)	102 (19.5)	522 (85.7)
Positive for CT and/or NG	63 (78.7)	17 (21.3)	80 (13.1)
Missing	6 (85.7)	1 (14.3)	7 (1.2)

AI, anal intercourse; AUDIT, Alcohol Use Disorders Identification Test; CECA, Childhood Experiences of Care and Abuse; DAST, Drug Abuse Screening Test; IQR, interquartile range; MOS‐SS, Medical Outcomes Study‐Social Support scale; PHQ‐9, Personal Health Questionnaire‐9; USAID HPI, United States Agency for International Development Health Policy Initiative.

^a^ Row percentages are presented. Row percentages may not equal 100.0% due to rounding

^b^ Column percentages are presented. Column percentages may not equal 100.0% due to rounding.

### 3.2 Retention outcome

Of the 609 men included in the primary analysis, 20.0%, 21.5%, 22.0% and 17.9% missed their months 3, 6, 9 and 12 follow‐up visits respectively. Overall, 8.5% missed all four study visits; 4.8% missed three study visits; 8.5% missed 2 visits and 15.9% missed one visit. Regarding the retention outcome of interest, 19.7% (95% CI (confidence interval): 16.6% to 23.1%) men missed two consecutive follow‐up visits (Table 1). There were missing results in some categories including; Resided in Kisumu for less than one year; Usual sexual position during sex with a male partner; Experienced recent trauma due to same‐sex behaviours; Experienced sexual or physical abuse during childhood; Harmful alcohol use and STI status. These missing results we generally less than 5% of the reported results thus are still generalizable.

### 3.3 Crude and adjusted regression analyses

In bivariate analyses (Table 2), missing two consecutive follow‐up visits was associated (*p* ≤ 0.20) with shorter length of residence in the study area at enrolment (<1 year vs. ≥1 year: OR, 3.62), not living with a male sexual partner (OR, 1.51), transactional sex during the last three months (OR, 1.35) and history of physical or sexual abuse during childhood (OR, 1.76). In the multivariable model, the odds of missing two consecutive follow‐up visits were increased for men who resided in the study area for <1 year (aOR, 4.14 [95% CI 1.77 to 9.68] *p* < 0.01), who were not living with a male sexual partner (aOR, 1.59 [95% CI 1.01 to 2.50] *p* 0.05) and who reported transactional sex during the last three months (aOR, 1.70 [95% CI 1.08 to 2.67] *p*‐ 0.02).

**Table 2:** Results of bivariate logistic regression: baseline characteristics of HIV‐negative GBMSM in Kenya associated with missing two consecutive study visits

Variable	OR (95% CI)	*p*‐value^a^	*p*‐value^b^
Age, years			
18 to 24	1.25 (0.83 to 1.89)	0.28	0.28
≥25	1.00 (ref)		
Education (years)			
0 to 8	1.36 (0.76 to 2.46)	0.30	0.49
9 to 12	1.30 (0.80 to 2.09)	0.29	
≥13	1.00 (ref)		
Enrolled in school			
Yes	0.82 (0.52 to 1.27)	0.37	0.37
No	1.00 (ref)		
Employment status			
Less than full‐time employed	1.18 (0.79 to 1.76)	0.41	0.41
Full‐time employed	1.00 (ref)		
Uncertain/very uncertain financial status			
Yes	0.86 (0.56 to 1.34)	0.51	0.51
No	1.00 (ref)		
Tribe/ethnicity			
Other	1.33 (0.81 to 2.19)	0.25	0.25
Luo	1.00 (ref)		
Resided in Kisumu for <1 year			
Yes	**3.62 (1.58 to 8.31)**	**<0.01**	**<0.01**
No	**1.00 (ref)**		
Any religious affiliation			
No	0.93 (0.42 to 2.05)	0.85	0.85
Yes	1.00 (ref)		
Marital status			
Married or living with female partner	0.90 (0.46 to 1.75)	0.75	0.90
Separated or divorced from female partner	0.90 (0.51 to 1.60)	0.72	
Single	1.00 (ref)		
Gay or homosexual sexual identity			
No	1.14 (0.75 to 1.75)	0.54	0.54
Yes	1.00 (ref)		
Currently living with a male sexual partner			
No	**1.51 (0.98 to 2.34)**	**0.06**	**0.06**
Yes	**1.00 (ref)**		
Transactional sex (participant received money, food, or housing) during the last three months			
Yes	**1.35 (0.88** to **2.06)**	**0.17**	**0.17**
No	**1.00 (ref)**		
Ever had sex with a female partner			
Yes	1.18 (0.75 to 1.86)	0.48	0.48
No	1.00 (ref)		
Always uses condoms during AI with a male sexual partner (last three months)			
No	1.14 (0.75 to 1.72)	0.55	0.55
Yes	1.00 (ref)		
Usual sexual position during sex with a male partner			
Receptive or versatile	0.91 (0.60 to 1.36)	0.64	0.66
Insertive	1.00 (ref)		
Experienced recent trauma due to same‐sex behaviours (last two weeks) (USAID HPI)?			
Yes	1.07 (0.70 to 1.63)	0.76	0.76
No	1.00 (ref)		
Experienced sexual or physical abuse during childhood (CECA)			
Yes	**1.76 (0.99 to 3.11)**	**0.05**	**0.05**
No	**1.00 (ref)**		
Harmful alcohol use (AUDIT ≥ 8)			
Yes	0.90 (0.60 to 1.35)	0.61	0.61
No	1.00 (ref)		
Harmful substance use (DAST ≥ 3)			
Yes	1.10 (0.69 to 1.74)	0.70	0.70
No	1.00 (ref)		
Any injection drug use in last year			
Yes	0.78 (0.32 to 1.91)	0.58	0.58
No	1.00 (ref)		
Social support (MOS‐SS)	1.00 (0.99 to 1.01)	0.76	0.65^c^
Moderately severe or severe depressive symptoms (PHQ‐9 ≥ 15)			
Yes	1.28 (0.69 to 2.38)	0.43	0.43
No	1.00 (ref)		
Circumcision status			
No	0.76 (0.46 to 1.24)	0.28	0.27
Yes	1.00 (ref)		
STI status			
Positive for CT and/or NG	1.11 (0.62 to 1.98)	0.72	0.72
Negative for CT and/or NG	1.00 (ref)		

AI, anal intercourse; AUDIT, Alcohol Use Disorders Identification Test; CECA, Childhood Experiences of Care and Abuse; CI, confidence interval; DAST, Drug Abuse Screening Test; MOS‐SS, Medical Outcomes Study‐Social Support scale; OR, odds ratio; PHQ‐9, Personal Health Questionnaire‐9; USAID HPI, United States Agency for International Development Health Policy Initiative.

^a^ *p*‐value is the result of the Wald chi square test from the bivariable logistic regression

^b^ *p*‐value is the result of the Pearson chi square test from contingency table analysis

^c^ *p*‐value is the result of the Wilcoxon rank sum test for continuous variables.

The bold values are the values that were significant from the analysis.

While the retention rates achieved in this study are not optimal, they are somewhat better than the limited estimates available from other studies of GBMSM in sub‐Saharan Africa. In a study of 449 HIV‐negative GBMSM followed in coastal Kenya, 25.7% did not report to the clinic within six months of the last planned study visit [12]. In a study of 441 HIV‐negative GBMSM in Nigeria, just 48.5% of participants attended their 12‐month visit [13], and among 327 HIV‐negative GBMSM recruited from Cape Town, Nairobi and Kilifi, attrition rates were 21.8 per 100 person‐years [14].

In our study, residing in the study area for <1 year, which was a baseline measure of length of residence, had the strongest association with missing two consecutive follow‐up visits. The immediate period following migration may be characterized by instability and lack of social support systems, including family members, friends, sexual partners and peer support groups [15, 16]. The men might also be returning to their previous area of residence and receiving services there. HIV prevention programmes should consider identifying individuals who are newcomers to the study area, assess family and social support and link them to local prevention and support services in a timely manner. A navigator system linking such men to a permanent resident peer may be helpful, as might be proactively asking recent arrivals about upcoming travel plans to link him with prevention services outside of the study area.

Similar to other studies [17, 18] among GBMSM in sub‐Saharan Africa, men who reported transactional sex had 70% increased odds of missing two consecutive visits. In Kenya, as in many other sub‐Saharan African countries, male same‐sex behaviours are criminalized and highly stigmatized [2, 3]. In our study, men who reported transactional sex more frequently experienced verbal insults, physical abuse, sexual abuse and verbal threats due to their perceived male‐sex behaviours [19] compared to men who did not report transactional sex (data not shown). Also, GBMSM who report transactional sex may have low retention rates due to psychosocial comorbidities including harmful alcohol use and severe depressive symptoms [19], both of which were more common among men who reported transactional sex (data not shown). Programmes to address these needs are urgently needed.

The odds of missing two consecutive follow‐up visits were increased for men who reported not living with a male sex partner. Only a small portion of men reported openly discussing their male same‐sex behaviours with family members (9.3%) or friends (12.0%). Thus, support from male sex partners may serve as an important buffer for GBMSM against limited interpersonal support regarding same‐sex behaviours. Support by male sex partners may be actively strengthened by programmes to help remind participants about appointments, provide financial assistance and facilitate greater engagement in HIV prevention programmes.

Although our data were collected in the context of a research study between 2015 and 2017, the experiences of the staff and participants in the *Anza Mapema* study may help future HIV prevention programmes anticipate challenges regarding the retention of GBMSM in rights‐constrained settings. In our study, staff implemented extensive retention procedures, not least of which was ensuring a safe, GBMSM affirming environment with nearly daily group activities to encourage engagement. Also, participants were reimbursed for their travel to the study clinic and compensated for their time at all scheduled follow‐up visits. Retaining GBMSM in prevention programmes is especially challenging, and retention may be even lower than observed here if significant effort and resources are not allocated to support intensive retention strategies and reasonable participant compensation.

The participants in this study may not be representative of GBMSM in Kisumu or Kenya since we used non‐probability sampling techniques to recruit participants. We collected data via ACASI, which has been shown to reduce response bias and interviewer bias [20, 21]. However, misreporting is still possible. The psychosocial scales we used have not been validated specifically among Kenyan GBMSM. However, many of the scales demonstrated acceptable internal reliability (Cronbach’s alpha ≥ 0.70) in the study [10]. We also did not collect information on procedures used to increase retention (number of reminders, supporting transportation fees for relocated individuals, physical tracing) and could not assess their association with retention.

## 4 CONCLUSIONS

The *Anza Mapema* study implemented comprehensive retention strategies that incorporated community engagement and staff capacity building, that created a GBMSM welcoming environment, and that incorporated participant tracing via multiple modalities. Despite these efforts, 19.7% of men missed two consecutive follow‐up visits, and the proportion of men missing any scheduled visit during the 12‐month study ranged from 17.9% to 22.0%. Support and involvement of male sexual partners with whom participants live may be a means to improve retention in HIV prevention programmes. For participants who have resided in an area for <1 year, retention may be improved if clinicians and counsellors emphasize available support services, promote social networking and peer support and facilitate engagement in HIV prevention services if participants decide to travel or locate elsewhere.

## COMPETING INTERESTS

All authors declare no conflict of interest in this manuscript and the work associated with it.

## AUTHORS’ CONTRIBUTIONS

FO, RCB, SDM, SMG, BON and GD carried out conception or design of the work – DO, FO, SDM, CK, SMG, RCB involved in acquisition, analysis or interpretation of data for the work. CK, RCB, SDM, FO, DO and SMG drafting the work. CK, RCB, DO, FO, SDM, SMG BON and GD revising the work critically for important intellectual content. All Authors read and approved the manuscript.
